# Effects of human parvovirus B19 VP1 unique region protein on macrophage responses

**DOI:** 10.1186/1423-0127-16-13

**Published:** 2009-01-24

**Authors:** Bor-Show Tzang, Chun-Ching Chiu, Chun-Chou Tsai, Yi-Ju Lee, I-Jung Lu, Jing-Yu Shi, Tsai-Ching Hsu

**Affiliations:** 1 Institute of Biochemistry and Biotechnology, Chung Shan Medical University, Taichung, Taiwan; 2 Institute of Immunology, Chung Shan Medical University, Taichung, Taiwan; 3 Department of Neurology and Department of Medical Intensive Care Unit, Chunghua Christian Hospital, Chunghua, Taiwan; 4 Department of Sports, Health & Leisure, Chihlee Institute of Technology, Taipei County, Taiwan

## Abstract

**Background:**

Activity of secreted phospholipase A (sPLA2) has been implicated in a wide range of cellular responses. However, little is known about the function of human parvovirus B19-VP1 unique region (VP1u) with sPLA2 activity on macrophage.

**Methods:**

To investigate the roles of B19-VP1u in response to macrophage, phospholipase A2 activity, cell migration assay, phagocytosis activity, metalloproteinase assay, RT-PCR and immunoblotting were performed.

**Results:**

In the present study, we report that migration, phagocytosis, IL-6, IL-1β mRNA, and MMP9 activity are significantly increased in RAW264.7 cells by B19-VP1u protein with sPLA2 activity, but not by B19-VP1uD175A protein that is mutated and lacks sPLA2 activity. Additionally, significant increases of phosphorylated ERK1/2 and JNK proteins were detected in macrophages that were treated with B19-VP1u protein, but not when they were treated with B19-VP1uD175A protein.

**Conclusion:**

Taken together, our experimental results suggest that B19-VP1u with sPLA2 activity affects production of IL-6, IL-1β mRNA, and MMP9 activity, possibly through the involvement of ERK1/2 and JNK signaling pathways. These findings could provide clues in understanding the role of B19-VP1u and its sPLA2 enzymatic activity in B19 infection and B19-related diseases.

## Background

Human parvovirus B19 (B19) is a human-pathogenic parvovirus consisting of a small non-enveloped particle with a single-stranded linear 5.6-kb DNA genome [1,2]. The icosahedral capsid consists of two structural proteins, VP1 (83 kDa) and VP2 (58 kDa), which are identical with the exception of 227 amino acids at the amino-terminal end of the VP1-protein, the so-called VP1-unique region (VP1u) [3]. Although VP2 proteins predominate in the capsid of B19, VP1 is critical in eliciting an appropriate immune response in both human and animal [4-6]. Previous studies have shown that antiserum produced by immunizing rabbits with a fusion protein containing the entire unique region sequence of VP1 neutralized the binding activity of B19 [7]. Recently, a phospholipase A2 (PLA2) motif has been linked to the B19-VP1u [8-11] and mutation of B19-VP1u in the phospholipase domain causes a complete loss in enzymatic activity and viral infectivity (ex: D175A) [10-12]. Although parvovirus PLA2 has been classified as a group XIII PLA2 [10,13], the precise function of secreted phospholipases A (sPLA2) from B19-VP1u is still obscure.

Macrophages are part of the innate immune system and are derived from monocytes, which develop in the bone marrow and play important roles in providing the first line of host defense [14]. The migration of macrophages through peripheral tissues is an essential step in the host response to infection and inflammation [15,16]. Additionally, pro-inflammatory cytokines are important mediators in the inflammatory process and are associated with the activation of macrophages, which leads to altered morphology and functional activity [15-17]. Although various cytokines are elevated in serum from patients with B19 infection and have been associated with the regulation of macrophages [17-19], the role of B19 VP1u on induction of cytokines in macrophages is still obscure.

Activity of sPLA2 has been implicated in a wide range of cellular responses, including cell proliferation, signal transduction, host defense, and chemokinesis [20-22], which includes extracellular matrix (ECM) remodeling [23-26], production of ECM proteins, and the activity of ECM homeostatic enzymes such as matrix metalloproteinase (MMP) and tissue inhibitor of metalloproteinase (TIMP). Previous studies have reported that PLA2 promotes dramatic migration and ECM invasion by NIH3T3, mouse fibrosarcoma, and sarcoma cells, via its high affinity receptor [22], which induces cell migration and MMP-2 activation via phosphoinositide 3-kinase (PI3K) and Akt [24,25]. These results indicate an association between sPLA2 and MMP activation. In our current study, we evaluated the enzymatic activity of recombinant B19-VP1u with PLA2 motif proteins and its effect on macrophages.

## Methods

### Preparation of recombinant wild type and mutated human B19 VP1 unique proteins

E. coli (BL21-DE3) clones containing VP1u or VP1uD175A cDNA (D175A proteins encoded by genes with point mutations in the enzyme catalytic site) in pET-32a expression vector (Novagene, Cambridge, MA) were obtained as described in our previous work that were used to produce the rabbit anti-B19-VP1u antibody [27-29].

### sPLA2 activity

B19-VP1u and B19-VP1uD175A proteins were assayed for sPLA2 activity by use of a colorimetric assay (sPLA2 Activity Kit; Cayman Chemical), in accordance with the manufacturer's instruction. Results are expressed as micromoles per minute per milliliter. Additionally, the incubation of B19-VP1u or B19-VP1uD175A proteins with rabbit anti-B19-VP1u, anti-B19-NS1 or normal rabbit IgG [27-29] was also assayed for sPLA2 activity.

### Cell culture

Mouse macrophages RAW 264.7 cells (RAW 264.7) were originally obtained from American type culture collection (ATCC) (Manassas, Va, USA) and were cultured in Dulbecco's modified Eagle medium (DMEM) supplemented with 10% (v/v) fetal bovine serum at 37°C and 5% CO2 incubator. Inhibitors of MAPK signal pathway were used to verify the expression ERK1/2 or JNK protein in RAW 264.7 cells. RAW 264.7 cells were incubated with 400 ng of B19-VP1u recombinant protein in the absence or presence of 10 μM U0126, a ERK inhibitor, or 25 μM SP600125, a JNK inhibitor, (SABiosciences, Co. MD. USA), for 24 h at 37°C, 5% CO2. The cell lysate were then obtained and stored at -80°C for further use.

### Migration assay

To determine the effect of VP1u or VP1uD175A on cell motility, cells were seeded into a Boyden chamber (Neuro Probe, Cabin John, MD) on membrane filters, and migration of cells stimulated or un-stimulated with VP1u, VP1uD175A, or VP1u+U0126 was measured as described. The sPLA2 from bee venom (bvPLA2; Sigma-Aldrich) was used as a positive control. A modified Boyden chamber assay using cell culture inserts with a 8-mm pore size polycarbonate filter in a 48-well format was used to perform an in vitro migration assay. Cells were seeded on the upper part of the chamber at a density of 2 × 10^5^ cells/well in 50 uL of serum free medium and then incubated for 16 h at 37°C. The bottom chamber contained standard medium with 10% FBS. The cells that had invaded to the lower surface of the membrane were fixed with methanol, washed with dd-H2O, and then stained with Giemsa (Sigma). Ten random fields were counted for each experiment under a light microscope.

### Assessment of phagocytosis

For phagocytosis of Latex beads, 2 × 10^5^ of RAW264.7 cells were cultured in each well of a 16-well Lab-Tek^®^II Chamber Slide™ (Nunc, Denmark) overnight and then stimulated with 400 ng of VP1u or VP1uD175A recombinant proteins for 16 h before incubation with FITC-labelled Latex beads (Sigma, Saint Louis Mo, USA). 100× FITC-labelled Latex beads were suspended in phosphate buffered saline (PBS) and opsonized by incubation with RAW264.7 cells for 4 h at 37°C. One hundred macrophages in five random fields were counted by observation under a light microscope, ZEISS AXioskop2 at a magnification of 200×. The phagocytic index was expressed as the number of phagocytosed particles divided by the total number of macrophages and expressed as a percentage [30].

### Messenger RNA isolation and RT-PCR

All studies were carried out in a designated PCR-clean area. After treatments at 0–24 h, washed in Dulbecco's phosphate buffered saline (DPBS) twice and treated with Trizol buffer for total RNA extraction. The cDNAs encoding mouse IL-1β, IL-6, TNFα, and GAPDH were amplified according to our previous condition [31] and the specific RNA level of every sample was expressed as the product's intensity. cDNA encoding glyceraldehyde- 3-phosphate dehydrogenase (GAPDH) was amplified and quantified for each sample.

### Gel zymography

RAW 264.7 cells were stimulated with VP1u/VP1uD175A recombinant proteins and the activities of MMP-2 and MMP-9 in medium were measured by gelatin-zymography assays as previously described [32]. Ten microliters of ten-fold diluted serum or 20 μl supernatant were separated by an 8% sodium dodecyl sulfate-polyacrylamide gel electrophoresis (SDS-PAGE) gels containing 0.1% gelatin. Gels were washed for 30 min in 2.5% Triton X-100 to remove the SDS and then soaked in the reaction buffer containing 40 mM Tris-HCl, pH8.0, 10 mM CaCl2 and 0.02% NaN3 for 30 min. Gelatinolytic activity was visualized by staining the gels with 0.5% Coomassie brillant blue R-250, de-stained with methanol-acetic acid water, and relative MMP levels were quantitated by a gel documentation and analysis system (AlphaImager, 2000, Alpha Innotech Corporation).

### Immunoblotting

Protein samples were separated in 12.5 or 10% of SDS-PAGE and electrophoretically transferred to nitrocellulose membrane (Amersham Biosciences, Piscataway, NJ, USA) according to the method of Towbin [33]. After blocking with 5% non-fat dry milk in (PBS), antibodies against AKT-p, Erk1/2-p, p38-p, JNK-p and NF-κB (p65-p), and actin (Upstates, Charlottesville, Virginia, USA) were diluted in PBS with 2.5% BSA and incubated for 1.5 hr with gentle agitation at room temperature. The membranes were then incubated with horseradish peroxidase (HRP) conjugated secondary antibody. Pierce's Supersignal West Dura HRP Detection Kit (Pierce Biotechnology Inc., Rockford, IL) was used to detect the antigen-antibody complexes. The blots were scanned and quantified by densitometry (Appraise, Beckman-Coulter, Brea, California, USA).

### Statistical analyses

The paired t test and one-way ANOVA were used to analyze for statistical significance. A P value < 0.05 was considered significant.

## Results

### Recombinant B19-VP1u proteins reveal sPLA2 activity

Recent studies have demonstrated that B19-VP1u possesses phospholipase A2 (PLA2) activity that is postulated to be involved in a variety of inflammatory reactions [8-11]. To confirm whether B19-VP1u protein has the PLA2 activity, the recombinant B19-VP1u protein and B19-VP1uD175A protein, a mutant form of B19-VP1u losing the PLA2 activity, were purified and analyzed for sPLA2 activity. Table 1 revealed the result of sPLA2 activity. No sPLA2 activity was detected in B19-VP1uD175A as previously shown [11]. Notably, apparent sPLA2 activity was detected in the recombinant B19-VP1u protein with an activity of 0.19 ± 0.03 μmol/min/mL, as well as bvPLA2 which is considered as a positive control with an activity of 0.56 ± 0.12 μmol/min/mL. As expected, significantly reduced sPLA2 activity was observed in recombinant B19-VP1u protein that was co-incubated with 5 ug of purified rabbit anti-B19-VP1u IgG antibodies.

**Table 1:** Secreted phospholipase A2 (sPLA2) activity of recombinant wild-type or mutated VP1-unique region (VP1u) proteins

Proteins	sPLA2 activity (μmol/min/mL)
bvPLA2 (1 ng)	0.56 ± 0.12
VP1u (0.5 ug)	0.03 ± 0.02
VP1u (5 ug)	0.19 ± 0.03 *^,&^
VP1uD175A(0.5 ug)	ND
VP1uD175A (5 ug)	ND
Rabbit anti-VP1u IgG (0.5 ug)	ND
Rabbit anti-VP1u IgG (5 ug)	ND
Rabbit anti-VP1uD175A IgG (0.5 ug)	ND
Rabbit anti-VP1uD175A IgG (5 ug)	ND
Rabbit IgG (0.5 ug)	ND
Rabbit IgG (5 ug)	ND
Rabbit anti-NS1 IgG (0.5 ug)	ND
Rabbit anti-NS1 IgG (5 ug)	ND
VP1u (5 ug) + Rabbit IgG (0.5 ug)	0.18 ± 0.07
VP1u (5 ug) + Rabbit IgG (5 ug)	0.19 ± 0.06
VP1u (5 ug) + Rabbit anti-B19-VP1u IgG (0.5 ug)	0.20 ± 0.11
VP1u (5 ug) + Rabbit anti-B19-VP1u IgG (5 ug)	0.09 ± 0.03 ^#^
VP1u (5 ug) + Rabbit anti-B19-VP1uD175A IgG (0.5 ug)	0.18 ± 0.05
VP1u (5 ug) + Rabbit anti-B19-VP1uD175A IgG (5 ug)	0.16 ± 0.06
VP1u (5 ug) + Rabbit anti-B19-NS1 IgG (5 ug)	0.16 ± 0.04
VP1uD175A (5 ug) + Rabbit anti-B19-VP1u IgG (5 ug)	ND
VP1uD175A (5 ug) + Rabbit anti-B19-VP1uD175A IgG (5 ug)	ND

bvPLA2: sPLA2 from bee venom; D175A proteins encoded by genes with point mutations in the enzyme catalytic site

ND means non-detected

* and ^&^indicate P < 0.05, compared to VP1u (0.5 ug) or VP1uD175A (5 ug), respectively.

^# ^indicates P < 0.05, compared to VP1u (5 ug).

### B19-VP1u induces migration and phagocytosis

**Figure 1:**
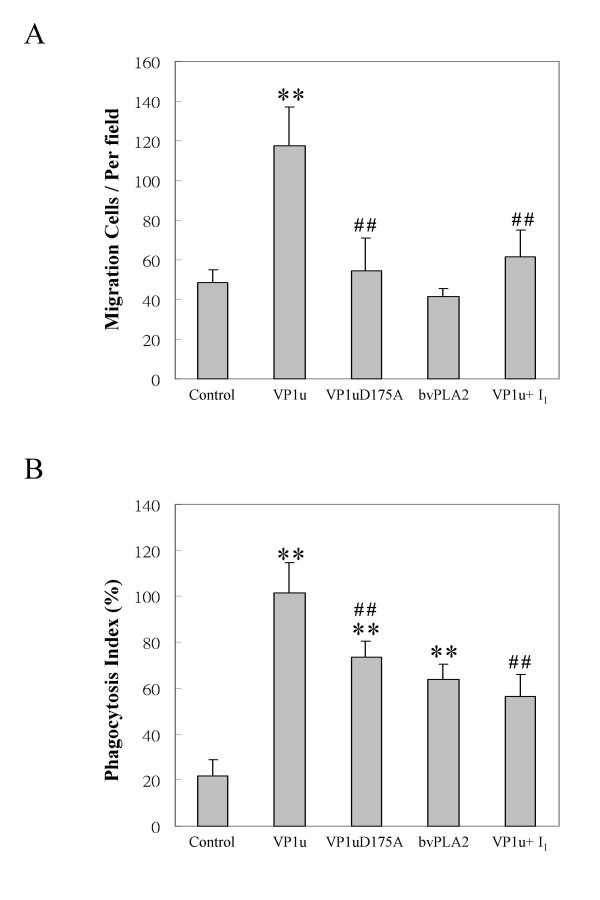
**Migration and phagocytosis of mouse Raw264.7 cells. The cells were incubated with different treatments in the upper compartment of Boyden chamber for 16 h**. (A) The migration of cells was determined by counting the number of migrated cells in 10 random fields of each well. (B) Phagocytic index of Raw264.7 cells was determined by pre-incubated with different preparations for 6 h before addition of FITC-labeled Latex beads. The cells with phogocytic FITC-labeled Latex beads were counted in 10 random fields. Similar results were obtained in three independent experiments. I_1 _indicates U0126. ** and ## indicate significant differences as compared to the control or the B19-VP1u group, respectively.

To test the effect of B19-VP1u on migration of macrophages, migration assays were performed as described in materials and methods. Figure 1A shows the results of migrated RAW264.7 cells through the membrane pores. No significant increase in migrated cells was detected in the VP1uD175A or bvPLA2 groups as compared to the control group. Notably, a significant increase of migrated macrophages was observed in the experimental group that was treated with B19-VP1u proteins as compared to the control group. In contrast, significantly decreased numbers of migrated macrophages were observed in the experimental group that was treated with B19-VP1uD175A or the B19-VP1u +U0126 as compared to the B19-VP1u group. To further examine the effect of B19-VP1u on phagocytosis, RAW264.7 cells were incubated under various treatments and co-cultured with FITC-labeled beads. The phagocytic ability was determined by calculating the phagocytic index (Figure 1B). Significant increases in the phagocytic index were detected in the B19-VP1u, B19-VP1uD175A, bvPLA2, and B19-VP1u+U0126 groups as compared to the control group. However, a significantly lower phagocytic index was detected in the B19-VP1uD175A and the B19-VP1u with U10126 groups as compared to the B19-VP1u group, respectively (Figure 1B).

### B19-VP1u induces IL-6, IL-1β mRNA expression and MMP9 activity

**Figure 2:**
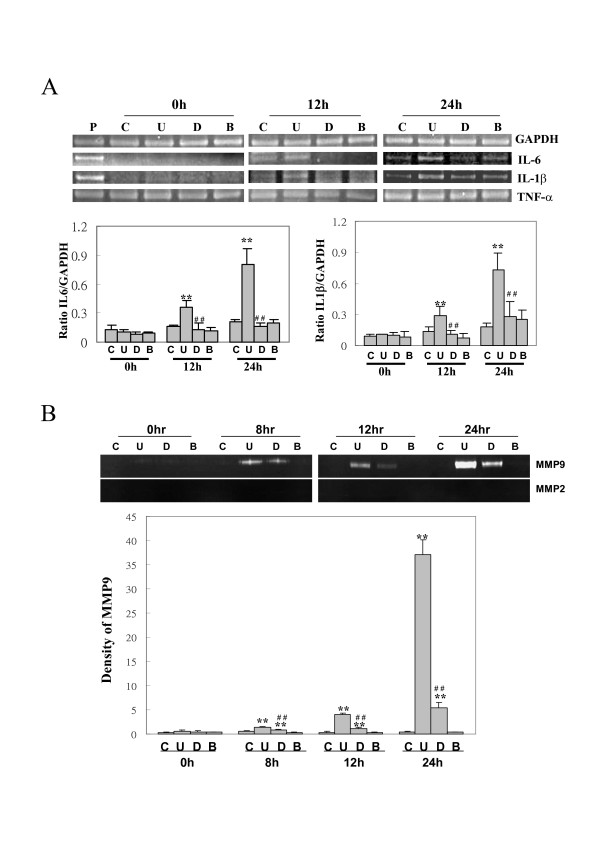
**Messenger RNA expression of various cytokines and MMP enzymatic activity in RAW264.7 cells**. (A) A representative agarose gel of RT-PCR for IL-6, IL-1β, and TNF-α. GAPDH is used as internal control. The lower panels are the quantitated results of IL-6 and IL-1β mRNA. (B) The activities of MMP9 and MMP2 were detected by zymographic assays and the quantified results are shown in the lower panels. Similar results were obtained in three independent experiments. C, U, D, B indicates experimental groups of control, B19-VP1u, B19VP1uD175A, and bvPLA2. ** and ## indicate significant differences as compared to the control or the VP1u group, respectively.

To examine the effect of B19-VP1u on expression of inflammatory cytokines in RAW264.7 cells, RT-PCR was performed. Figure 2A represents the RT-PCR results of IL-6 and IL-1β. Significant increases in IL-6 and IL-1β mRNA expression were observed in RAW264.7 cells that were stimulated with B19-VP1u at 12 and 24 h. In contrast, no induction of IL-6 and IL-1β mRNA expression was detected in cells that were stimulated with either B19-VP1uD175A or bvPLA2. Quantified results of IL-6 and IL-1β levels are shown in the lower panel of Figure 2A. To examine whether B19-VP1u and its sPLA2 enzymatic activity could influence the activities of the ECM degrading proteases such as MMP9 or MMP2, the effect of B19-VP1u on the secretion of MMPs was investigated using a zymography assay. As shown in Figure 2B, significant increases in MMP9 activity was observed at 8, 12 and 24 h in the experimental group treated with B19-VP1u proteins as compared to the control group. However, significant decreases in MMP9 activity were observed in the experimental group that was treated with B19-VP1uD175A at 8, 12 and 24 h as compared to the B19-VP1u group. Quantified results are shown in the lower panel of Fig 2B.

### B19-VP1u increases the phosphorylation of ERK 1/2 and JNK

**Figure 3:**
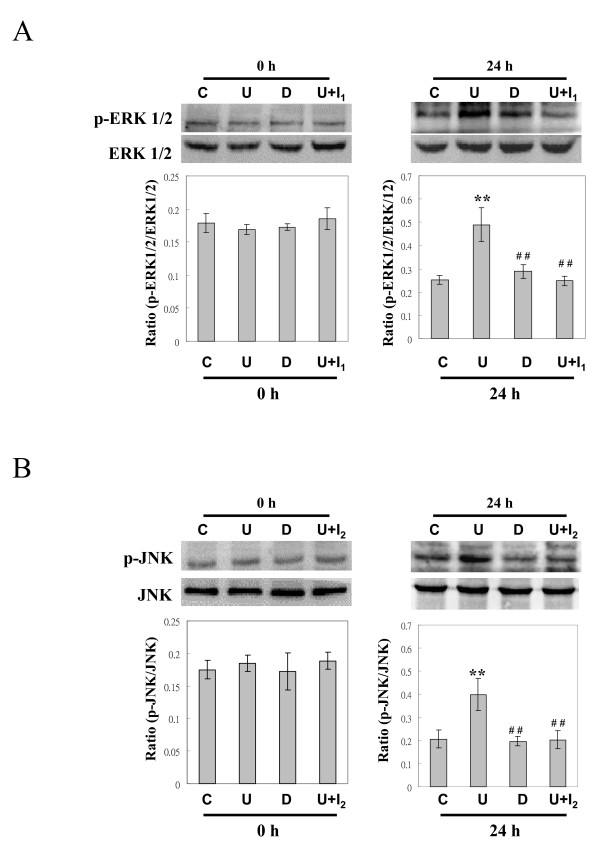
**Increased phosphorylation of ERK1/2 and JNK proteins in RAW264.7 cells pre-incubated with B19-VP1u**. The representative immunoblotting results for (A) ERK1/2 and (B) JNK proteins hybridized with mouse antibodies against phosphorylated -ERK1/2 and -JNK. The quantified results are shown in the lower panel. Similar results were obtained in three independent experiments. C, U, D, I_1_, and I_2 _indicate the experimental groups of control, B19-VP1u, B19VP1uD175A, U0126, and SP600125. ** and ## indicate significant differences as compared to the control or the VP1u group, respectively.

To clarify the possible signaling pathways involved in the activation of macrophages by B19-VP1u, various signaling molecules including AKT-p, Erk1/2-p, p38-p, JNK-p and NF-κB (p65 -p) were examined. Notably, the phosphorylation of ERK 1/2 and JNK proteins was significantly increased in RAW264.7 cells that were stimulated with B19-VP1u as compared to those stimulated with B19-VP1uD175A or control (Figure 3A and 3B). Additionally, significant decreases of phosphorylated ERK 1/2 and JNK proteins were observed in the presence of U0126, an ERK inhibitor, or SP600125, a JNK inhibitor (Figure 3A and 3B). However, no significant variations of AKT-p, p38-p, and NF-κB (p65-p), were observed in all experimental groups (data not shown). Quantified results are shown in the lower panels of Fig 3A and 3B.

## Discussion

Human parvovirus B19-VP1u is known to have a phospholipase A2 (PLA2) motif [8-12] and its enzyme activity has been associated with various inflammatory processes that may contribute to the activation of macrophages. However, little is known about the effects of B19-VP1u on macrophage activation. This is the first study to demonstrate the role of B19-VP1u and its sPLA2 enzymatic activity on the induction of macrophage responses that may contribute to inflammatory processes or diseases. Our results reveal the sPLA2 activity of recombinant B19-VP1u proteins can activate macrophages by significantly increasing migration and phagocytosis. Additionally, significant increases of MMP9 activity, IL-6 and IL-1b mRNA expression were detected in macrophages that were stimulated with B19-VP1u as well as the increased phosphorylation of ERK1/2 and JNK proteins.

Activity of sPLA2 has been implicated in a variety of physiological and pathological responses, including cell proliferation, chemokinesis [20-22], ECM remodeling [23-26,34], vascular inflammation, [35,36] and cerebral ischemia [37]. Recently, parvovirus B19-VP1u has been linked with sPLA2-like activity that is recognized as group XIII enzyme [10,13], a novel type of sPLA2 that may contribute to various pathological processes [8,26,35-38]. Additionally, a B19-VP1u mutant, B19-VP1uD175A, is mutated in the phospholipase domain and loses its enzymatic activity and viral infectivity [8-13]. However, little is known about the sPLA2 enzymatic activity of B19-VP1u in migration and phogocytosis of macrophages that is associated with a variety of inflammatory and immune responses. Notably, our experimental results reveal significant increases in migration and phagocytosis in RAW264.7 macrophage cells treated with recombinant B19-VP1u proteins as compared to the control group. In contrast, no induction of migration and phagocytosis was observed in the experimental group that was treated with B19-VP1uD175A. These experimental results strongly suggest that the sPLA2 enzymatic activity of B19-VP1u is crucial to activation of migration and phagocytosis of macrophages.

Indeed, phospholipase A2 is known to play important roles in many inflammatory processes and immune responses including atherosclerosis, pro-inflammatory cytokine expression, and cerebral ischemia [35,36]. Recent studies have demonstrated the association between extracellular-matrix-related proteins and inflammatory responses via activation of mitogen activated kinases (MAPKs). In a cerebral-ischemic animal model, ischemia and organ culture were demonstrated to induce the activation of p38, ERK 1/2 and SAPK/JNK in cerebral arteries and the expression of inflammatory and MMP9 genes in the wall of cerebral arteries [24,25,34,35,37]. Additionally, increased secretion of CXCL8 by sPLA(2)-IB is related to activation of NF-kB in human neutrophils [38] Recently, various studies have postulated MMP9 may contribute to the development or pathogenesis of autoimmunity [39]. However, the roles of B19-VP1u and its sPLA2 enzymatic activity on B19-related diseases have not been investigated. Notably, a similar phenomenon was observed in our experimental results. We demonstrated an increase in MMP-9 activity, IL-6 and IL-1β mRNA expression, and phosphorylation of ERK1/2 and JNK proteins in RAW264.7 cells that were treated with recombinant B19-VP1u proteins. In contrast, no inductive effects on inflammatory related responses were observed in RAW264.7 cells treated with mutant B19-VP1uD175A protein or B19-VP1u with inhibitors. These experimental results strongly indicate the importance of sPLA2 activity of B19-VP1u in activation of macrophages and suggest the involvement of B19-VP1u and its PLA2 activity in inflammatory processes via activation of ERK1/2 and JNK signaling pathways in macrophages.

## Conclusion

Taken together, parvovirus B19-VP1u and its sPLA2 enzymatic activity are critical for eliciting macrophage responses associated with a variety of inflammatory processes. Our experimental results demonstrate the effects of sPLA2 activity in B19-VP1u proteins by increasing migration, phagocytosis, and inflammatory responses such as significant increases of MMP9 activity, IL-6 and IL-1β mRNA expression in macrophages. This study strongly suggests the crucial role of sPLA2 activity of B19-VP1u in macrophage activation and may provide clues in understanding the role of B19-VP1u in the host response to B19 infection and B19-related diseases.

## Competing interests

The authors declare that they have no competing interests.

## Authors' contributions

BST and CCC conceived this study, drafted the manuscript, and performed the performed statistical analyses. CCT performed the RT-PCR, zymography, and Western blotting. YJL and IJL provided material support and encouragement for this work. TCH provided material support and direction, and drafted significant portions of the manuscript.
